# Supplementary material for: Off-axis metasurfaces for folded flat optics
Source: Nat Commun. 2023 Sep 12;14:5602. doi: 10.1038/s41467-023-41123-x (PMC10497541; doi:10.1038/s41467-023-41123-x)
Supplement: Supplementary file 1 — Supplementary Information [file 41467_2023_41123_MOESM1_ESM.pdf]

# Off-axis Metasurfaces for Folded Flat Optics

Brandon Born[1], Sung-Hoon Lee[2], Jung-Hwan Song[1], Jeong Yub Lee[2], Woong Ko[2], Mark L. Brongersma[1,*]

[1] Geballe Laboratory for Advanced Materials, Stanford University, Stanford, California, USA

[2] Samsung Advanced Institute of Technology, Samsung Electronics Co. Ltd., Samsung-ro 130, Yeongtong-gu, Suwon-si, Gyeonggi-do 16678, South Korea

[*] Correspondence and requests for materials should be addressed to Mark L. Brongersma (email: brongersma@stanford.edu).

## Supplementary Figures

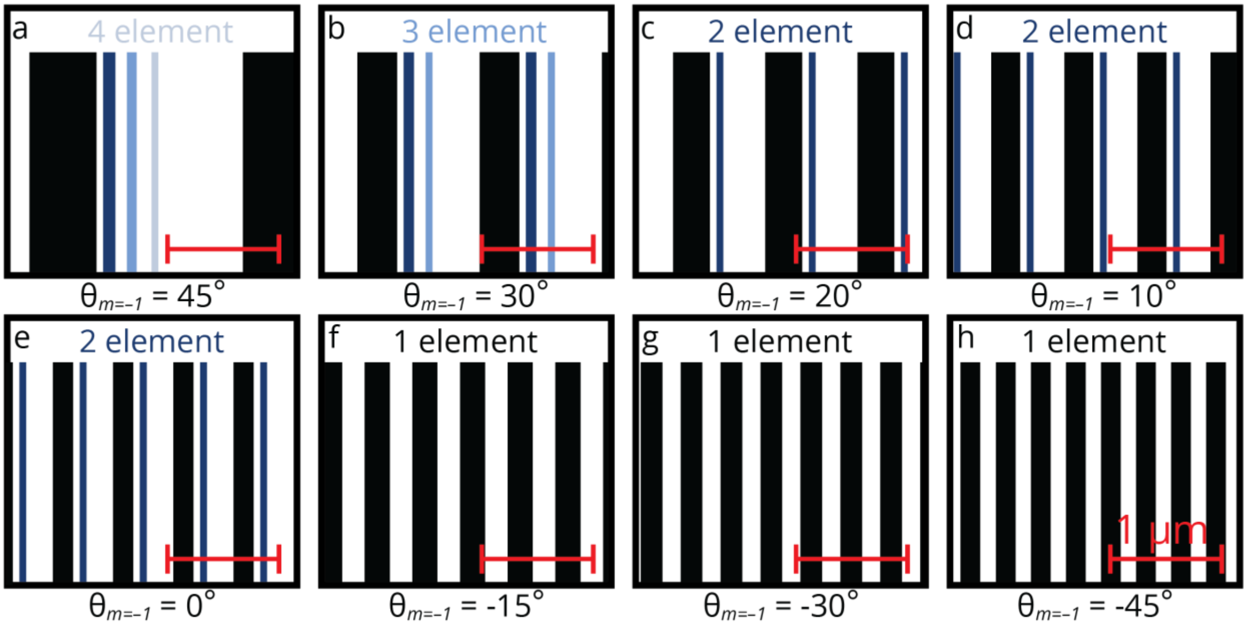


**Figure S1 | Metagrating dimensions for the eight experimentally validated samples from Figure 2d.** **a,** Each grating is optimized at the given exit angle, $\theta_{m=1}$, labelled under the image at $\lambda = 532$ nm and $\theta_i$ =80°. The dimension width and gaps for $\theta_{m=1}$ = +45° with a supercell pitch, $\Lambda$ = 1916 nm, are [602, 60, 110, 103, 87, 135, 60] rounded to the nearest nanometer. **b,** $\theta_{m=1}$ = +30° with $\Lambda$ = 1097 nm and widths = [356, 60, 92, 106, 60] nm. **c,** $\theta_{m=1}$ = +20° with $\Lambda$ = 828 nm and widths = [331, 60, 60] nm. **d,** $\theta_{m=1}$ = +10° with $\Lambda$ = 656 nm and widths = [260, 60, 60] nm. **e,** $\theta_{m=1}$ = 0° with $\Lambda$ = 540 nm and widths = [180, 60, 60] nm. **f,** $\theta_{m=1}$ = –15° with $\Lambda$ = 428 nm and a width = 225 nm. **g,** $\theta_{m=1}$ = – 30° with $\Lambda$ = 358 nm and a width = 194 nm. **h,** $\theta_{m=1}$ = –45° with $\Lambda$ = 314 nm and a width = 177 nm. The red scale bar is 1 μm.

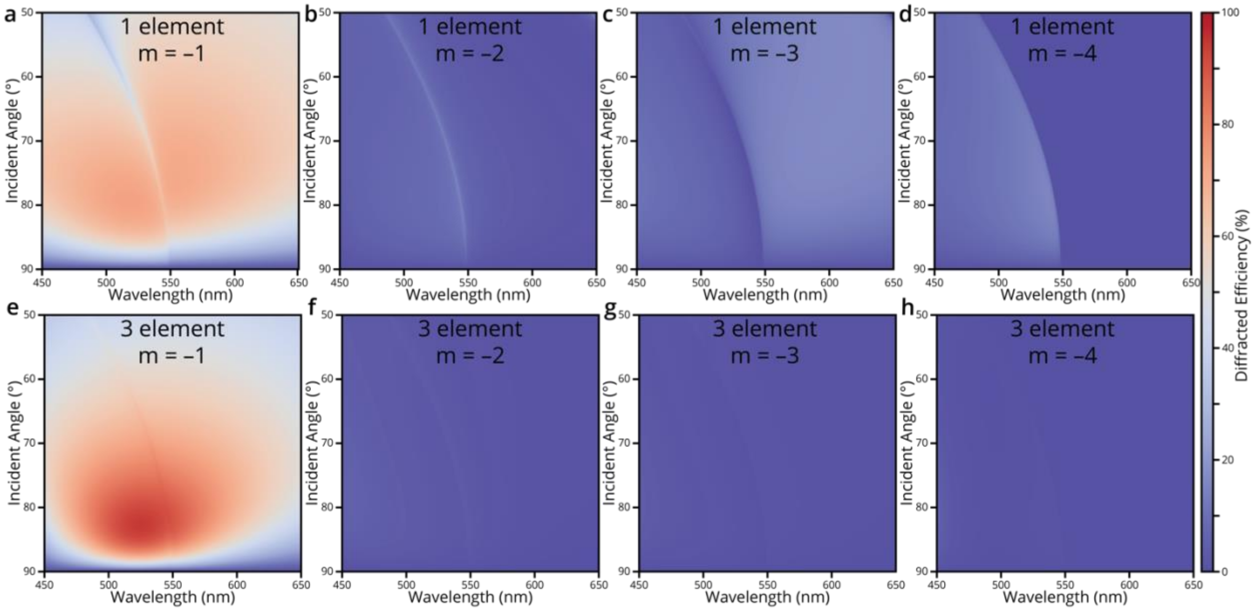


**Figure S2 | Diffracted efficiency comparison of 1-element and 3-element metagratings. a–d,** Diffracted efficiency for each diffraction order, $m = –1$ to –4, for a one-element metagrating, optimized to have a 45% duty cycle. The optimization conditions correspond to s-polarized light at $\theta_i = 80°$, $\theta_{m=-1} = +30°$, and $\lambda = 532$ nm. The power distributed to the higher orders is significant, with a sum of 25% at the optimized condition and 1% lost to specular reflection. Ultimately the first order efficiency is limited to 72%. **e–h,** Diffracted efficiency for each diffraction order, $m = –1$ to –4, for the three-element optimized metagrating depicted in Figure S1b and measured in Figure 2d. The predicted diffracted efficiency is 92% at the optimized condition, with the total higher order efficiency suppressed to 3% and 3% lost to specular reflection. The remaining power is lost to metal absorption.

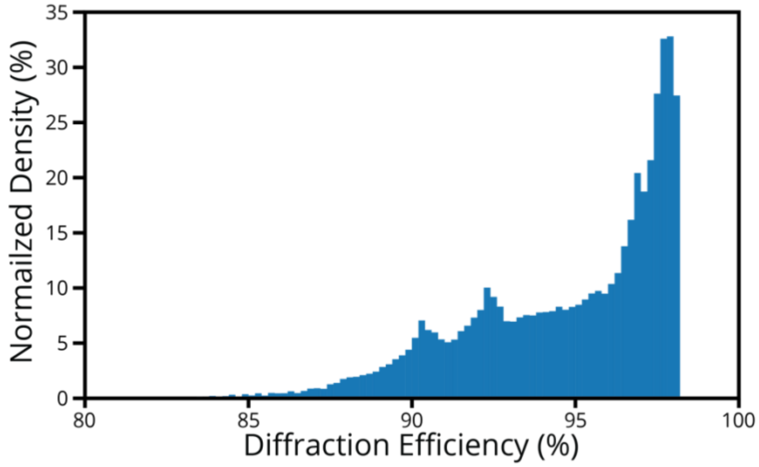


**Figure S3 | Histogram of the simulated diffraction efficiency from Figure 3b for the final design.** Efficiency ranges from 83% to 98%, with a mean of 95%. Efficiencies above 95% largely originate for the $x > 0$ half of the metasurface. The results show 92% of the metasurface area has diffraction efficiency above 90%.

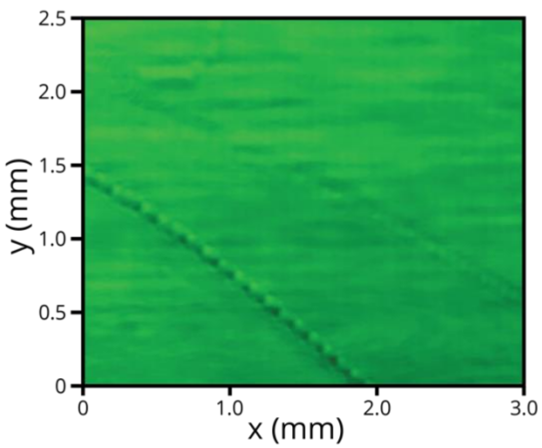

**Figure S4 | Magnified 50×50 μm pixel boundaries of the flat-field image from Figure 3d.** This image is captured by placing the camera's aperture at the metasurface focal point and has been corrected for distortion. The image is zoomed to 3.0×2.5 mm of the overall 2×2 cm metasurface size, and is centered at approximately $x =$ –6 mm, $y =$ +6 mm.

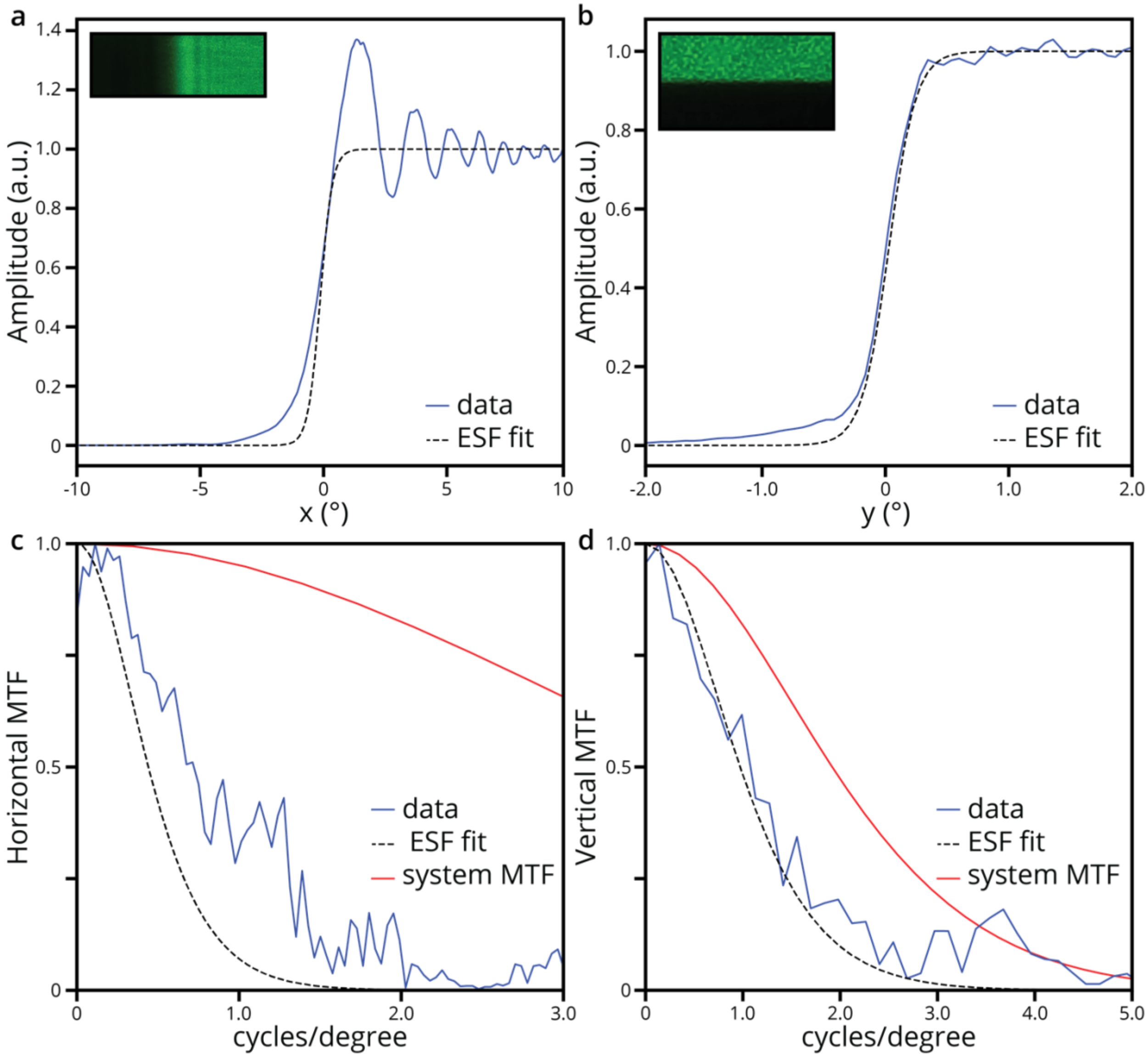


**Figure S5 | a,** Horizontal edge-gradient measurement of 2×2 $cm^2$ metasurface sample from Figure 4b. The intensity data is averaged perpendicular to the edge and fit with edge spread function (ESF). **b,** Vertical ESF measurement. **c,** Horizontal modulation transfer function (MTF) calculated from ESF data (blue line). The MTF fit (dashed black line) is calculated directly from the ESF fit in panel (a) and is 0.5 at approximately 0.4 cycles per degree. System MTF (red line) is calculated from a calibration image. **d,** Vertical MTF data (blue line) with ESF fit (dashed black line) and calibrated system MTF (red line). The MTF is at least 0.5 at 1 cycle per degree. The measured MTF is limited by the system MTF due to the measurement optics and mechanical constraints.

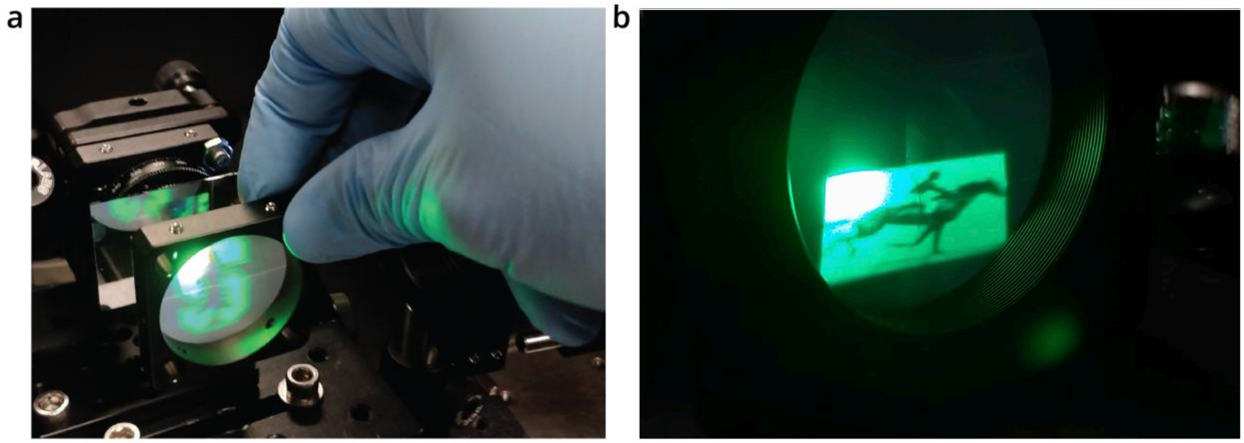

**Figure S6 | a,** Experimental setup used to capture moving images seen in panel b. The setup is the same as Figure 4a with the addition of an opaque screen placed at the projected image plane. The camera capturing this video recording is placed on the transmission side of the opaque screen. **b,** Demonstration of moving images projected with the 2×2 cm metasurface. Collimated light from a portable laser projector is used to play the first film of a racehorse, recorded in 1878 by Eadweard Muybridge.
